# HIV coreceptor tropism determination and mutational pattern identification

**DOI:** 10.1038/srep21280

**Published:** 2016-02-17

**Authors:** Hui-Shuang Shen, Jason Yin, Fei Leng, Rui-Fang Teng, Chao Xu, Xia-Yu Xia, Xian-Ming Pan

**Affiliations:** 1 The Key Laboratory of Bioinformatics, Ministry of Education, School of Life Sciences, Tsinghua University, China; 2 Department of Biostatistics, Saw Swee Hock School of Public Health, National University of Singapore, Singapore

## Abstract

In the early stages of infection, Human Immunodeficiency Virus Type 1 (HIV-1) generally selects CCR5 as the primary coreceptor for entering the host cell. As infection progresses, the virus evolves and may exhibit a coreceptor-switch to CXCR4. Accurate determination coreceptor usage and identification key mutational patterns associated tropism switch are essential for selection of appropriate therapies and understanding mechanism of coreceptor change. We developed a classifier composed of two coreceptor-specific weight matrices (CMs) based on a full-scale dataset. For this classifier, we found an AUC of 0.97, an accuracy of 95.21% and an MCC of 0.885 (sensitivity 92.92%; specificity 95.54%) in a ten-fold cross-validation, outperforming all other methods on an independent dataset (13% higher MCC value than geno2pheno and 15% higher MCC value than PSSM). A web server (http://spg.med.tsinghua.edu.cn/CM.html) based on our classifier was provided. Patterns of genetic mutations that occur along with coreceptor transitions were further identified based on the score of each sequence. Six pairs of one-AA mutational patterns and three pairs of two-AA mutational patterns were identified to associate with increasing propensity for X4 tropism. These mutational patterns offered new insights into the mechanism of coreceptor switch and aided in monitoring coreceptor switch.

AIDS has claimed millions of lives worldwide and continues to be a pressing public health issue. To combat HIV replication in an infected individual, anti-retroviral therapies (ARTs) have been designed to target essential proteins in the replication cycle of HIV. The replication cycle is comprised of several steps. To successfully enter a cell, the viral gp120 protein first binds to its primary cellular CD4 receptor protein. This induces a series of conformational changes in the gp120 protein, enabling the virus to bind either to the CCR5 or CXCR4 coreceptor^1^. After, virus-cell membrane fusion occurs and the viral entry process is complete. Based on the coreceptor type, HIV exhibits different tropisms. HIV usually requires CCR5 to facilitate primary infection^2^, but approximately half of infected individuals will switch to CXCR4 usage, which is generally associated with an accelerated decline in the CD4+ cell count and rapid disease progression^3^,^4^. Only one CCR5 inhibitor –Maraviroc – has been licensed for use in the antiretroviral treatment due to the severity of side effects from other cellular receptor blocker candidates^5^. Its long-term efficacy depends on the HIV’s utilization of the CCR5 coreceptor. Consequently, identifying the specific coreceptor usage is essential for effective drug intervention.

Viral coreceptor tropism can be determined *in vitro* by phenotypic assays^6^,^7^. However, due to complicated laboratory work and slow processing times, we need to develop simpler, faster, cheaper and more accessible *in silico* procedures to measure dynamic coreceptor usage. Genotypic tropism prediction methods are usually based on the third variable loop (V3)^8^,^9^ of the viral glycoprotein gp120, which has been identified as an important determinant for coreceptor usage^8^,^10^. The V3 loop is a highly variable sequence that includes amino acid (AA) deletions, insertions, and mutations, even a single amino acid change in V3 loop could switch the tropism^11^,^12^. Generally, CXCR4-tropic V3 sequences have a higher charge, a lower sequence identity (higher genetic diversity among CXCR4-tropic sequences), and a longer sequence^13^ from which genotypic models derive sequence features. The first model developed for inferring coreceptor usage is called the 11/25 rule, which classifies the virus as having CXCR4 tropism if a positively charged amino acid is observed in positions 11 or 25 of the V3 sequence^14^. This simple approach can be easily carried out, but has a low sensitivity (a CXCR4-tropic virus can be incorrectly characterized as CCR5-tropic), making it unsuitable for routine clinical use. More sophisticated methods have been developed, such as methods based on Support Vector Machine (SVMs)^15^,^16^,^17^, Position-Specific Weight Matrix (PSSM)^18^ and artificial neural networks^19^. Although these machine learning based methods yielded higher accuracies on coreceptor tropism identification compared to the 11/25 rule, they do not explore genetic mutations associated with tropism switch.

Here, we present a reliable model comprised of two coreceptor specific weight matrices (CMs). The tropism of a new V3 sequence is determined based on scores calculated from these two CMs. By grouping the sequences based on scores derived from CMs and performing a large-scale pattern analysis across these groups, we were able to identify key mutational pattern pairs involved in coreceptor switch. Our method couples the classification using CMs, incorporating charge rules, alongside mutational pattern search to better elucidate the tropism switch process.

## Methods

### Training Dataset

We extracted 6,205 purely CCR5-tropic and 1,685 mixed CXCR4-tropic (labeled with “CXCR4” or “CCR5 CXCR4”) V3 loop sequences of HIV-1 from the Los Alamos HIV sequence database (http://www.hiv.lanl.gov/, last modified 10 Dec 2014). According to the HIV database, coreceptor tropism information of each annotated sequence was based on the phenotypic assays and not on presumed usage inferred from sequences. A variety of traditional biological assays, including MT-2^20^, NP-2^21^, U87^22^, Ghost^23^ and U373-MAGI^24^ cell lines, and several commercial recombinant assays including Trofile^25^, Phenoscript^26^, Vicro^27^, TTT^28^, PhenXR and XtrackC^29^ have been used for determination of coreceptor usage. These phenotypic assays were treated as the gold standard for tropism determination, despite the sensitivity being affected by the minor X4 variants level. To avoid bias due to overrepresentation of data from repeated sequences, we removed all duplicate sequences. Sequences longer than 39 or shorter than 31 amino acids were also removed. Also, when developing a classifier, discordance between genotyped sequences and sequences effectively used in the phenotypic assay may be ambiguous and misleading. To bypass this, only clonally derived sequences were included when filtering the R5 × 4 dataset. This process (Figure S1) led to a dataset containing 2,354 R5-tropic (145 subtype A, 1,229 subtype B, 472 subtype C, 137 subtype D, 134 subtype 01_AE, 137 other subtypes) and 325 × 4-tropic (6 subtype A, 130 subtype B, 50 subtype C, 60 subtype D, 54 subtype 01_AE, 31 other subtypes) unique V3 sequences. For the model development, we aligned each of the sequences in the training set to the consensus sequence retrieved from the Los Alamos HIV sequence database using Needleman-Wunsch^30^ (Version: EMBOSS: 6.6.0.0) with default parameters. We obtained a dataset of sequences with the same length (35AA). Gaps in the aligned dataset, denoted by the letter B, were allowed. The resulting dataset used for the training and validation is provided in the additional files “Dataset 1” (R5) and “Dataset 2” (X4). Unless stated otherwise, we assumed the inclusion of 21 amino acids in the aligned dataset.

### Independent Dataset

An independent dataset is necessary to evaluate the performance of any newly developed method. To construct this independent dataset, we screened papers for possible V3 sequences with tropism information and filtered these sequences with our training dataset to keep them non-repetitive. In this way, we obtained an independent dataset (“Dataset 3” for R5 and “Dataset 4” for X4) containing 221 R5-tropic and 91 × 4-tropic V3 sequences from previous studies^15^,^31^,^32^,^33^,^34^,^35^.

### Coreceptor-Specific Weight Matrices

A total of 2,354 R5 and 325 × 4 aligned V3 sequences were used to construct R5 and X4 coreceptor-specific weight matrices (CMs, see the download page of webserver), respectively. Let us take the R5-specific weight matrix (R5-M) as an example. To avoid biases due to the different frequencies of the 21 amino acids, we first derived a “dividing factor”, F (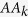), for each amino acid (Table S1):


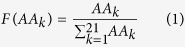


Here, 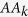 represents the count number of amino acid k (=1 to 21) in the R5 sequences dataset.

Because all aligned sequences have a length of 35 and consist of 21 letters (representing 20 amino acids, plus B for the gaps), this leads to a 35 × 21 matrix. The value Pij for rows i and column j is defined as:


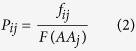


Where is given by:


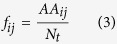


Here, 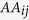 denotes the count number of the amino acid j (=1 to 21) in the ith (=1 to 35) position in the set of R5 sequences, and 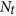 represents the total number of R5 sequences.

In theory, for a new V3 sequence with a fixed length, its distance from the R5 dataset can be scaled by multiplication with the corresponding probability P_ij_ for position i of amino acid j. However, we might obtain a value of zero because not every amino acid will appear in each position. We therefore took the logarithm of each entry and defined log0 = −3, which was optimized by 10-fold cross validation result. Then, the final score for the newly aligned V3 sequence for the R5 sequence dataset was converted to a sum of the 35 probability entries:


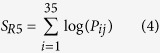


To date, several studies^12^,^14^,^36^,^37^,^38^,^39^,^40^ have reported a key role of the V3 charge in coreceptor tropism. Improved performance has been observed by incorporating the V3 charge rule (11/25 rule and V3 net charge). The 11/25 rule^14^ predicts a viral strain to be X4 when positively charged amino acids (K or R) are present at position 11 or 25 of the V3 loop. The presence of positively charged residues in specific positions is also associated with a net charge (NC) increase, which is already used in HIV-1 tropism prediction^41^. Hence, in this study we combined the weighting matrix method with the charge rule by two charge rule-related parameters (one for 11/25 rule and one for NC) when calculating the score for a newly aligned V3 sequences in X4 sequence dataset. The score in X4-M is given by


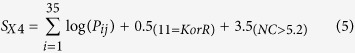


We optimized these two charge rule-related parameters based on the performance of ten-fold cross validation. Serial parameters for each charge rule-related item (position 11, position 24/25 and cut off value of NC) were tested to identify the best combination of parameters based on the results of cross validation. The second term implies that S_X4_ increases by an additional 0.5 when the amino acid in position 11 of a newly aligned V3 sequence is K or R. Based on the results of cross validation, worse performance was found only when we tried to assign any additional values for the positive charge on position 24/25. The last term takes the NC into account with a cut off value of 5.2. After optimization, we assigned an extra value of 3.5 for one sequence when the NC value of the sequence is above 5.2.

The final score for predicting the tropism of a new V3 sequence was given by


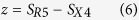


In the above equation and S_R5_ and S_X4_ are scores in R5-M and X4-M, respectively. If the formula’s output z is above zero, a new given V3 sequence would be classified as R5-tropic (Table S2, sheet R5). Otherwise, it would be classified as X4-tropic (Table S2, sheet X4). With the exception when calculating NC values, this required that all sequences had a length of 35.

### Validation

Compared to the R5 dataset, the X4 training dataset was much smaller, possibly causing an over-fitting problem in the X4 scoring matrix. To limit this problem and to gain insight into how these CM scoring models would generalize to an independent dataset, we performed a ten-fold cross validation study^42^,^43^ in which a random number between 0 and 9 was separately generated for each sequence in the R5 and X4 datasets to maintain uniform sampling. Sequences with the same number would be grouped into the same subset. One subset was used for testing, and the remaining subsets were used to obtain the R5- and X4-specific score matrices. Each subset could be used once for testing after ten repetitions, and the performances of these ten testing set results were combined to yield the final accuracy estimation.

### Evaluation

We used receiver-operator characteristic curves (ROCs) to illustrate the trade-off between specificity (true negative rate) and sensitivity (true positive rate) on the basis of the overall ability to discriminate between R5 and X4 sequences. The area under the curve (AUC)^44^ is a measure of the test’s ability to correctly predict the phenotype of any sequence: an area of 0.5 indicates a completely random guess, while an area of 1 shows a perfect test. We used the ROCR package^45^ in R for visualizing and calculating the AUC. Although there is no perfect way to evaluate prediction methods using a single number (e.g., accuracy), we used Matthew’s correlation coefficient (MCC) due to its robustness when dealing with different class sizes. The MCC was directly calculated using the following formula:


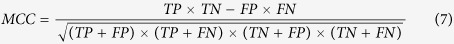


Here, TP is the number of true positives, which, in our case denotes the number of correctly predicted X4-tropic sequences. TN is the number of true negatives, FP is the number of false positives, and FN is the number of false negatives. An MCC value of ‘1’ corresponds to a perfect prediction, whereas a value of ‘0’ corresponds to a completely random prediction.

### Pattern Analysis

To identify sequence markers associated with coreceptor switch, we performed a systematic pattern search across the V3 region using one-AA and two-AA pattern pools separately. In the one-AA pattern pool, two kinds of information were used to denote a pattern symbol: a position number, and an AA character (e.g. 1C). Based on this, the one-AA pattern pool included 700 combinations (35 × 20) of all possible positions (35) and AAs (20). To generate the two-AA pattern pool, we fixed a 20 AA length window and randomly chose 2 AAs to occupy any 2 of 20 positions, such as ‘C….N……………’ (2 AAs and 18 blanks). The number of possible position combinations for 2 residues in a window of 20 spaces is 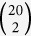, giving a total of 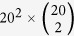 possible patterns. For brevity, we named this pattern set Pat20-2, which represented all possible patterns with 2 residues in a 20-length window. We also generated pattern sets Pat20-3 and Pat20-4 to repeat the pattern search procedure for validating changes resulting from any changes in the AA number. The pattern analysis was performed in two steps. The first step was to locate important positions and AAs crucial for coreceptor switch, and the second step was to derive corresponding “pattern pairs” associated with tropism switch.

We assumed that the patterns concentrated in the X4 dataset and not in the R5 dataset were crucial to tropism shift. To identify these X4-tropic patterns, all correctly predicted R5 and X4 sequences were clustered into eight groups (four clusters for R5 and four clusters for X4, see Table S3) based on their individual scores, all while keeping the group sizes and score intervals as balanced as possible among groups. After, all possible one-AA and two-AA patterns were used to screen all eight groups, yielding a pattern-count (the number of sequences that matched each pattern) per group. To calculate proportions of each pattern across the eight groups, we took the ratio between the pattern-count and the group size (count of all sequences in this group). Then, a student’s t-test for proportion values (four values in the R5 and X4 dataset) was performed for each pattern to determine if one pattern was significantly different between the R5 and X4 dataset (Table S4, Sheet1 and Sheet2). Patterns with a higher proportion average in X4 and a p-value below 0.05 were identified as X4-tropic patterns. From these patterns, we identified the crucial positions and AAs associated with coreceptor switch. To locate these positions, we used pairwise alignments to the consensus sequence. To identify AAs, the composition of AAs in these X4-tropic patterns and whole X4 dataset were calculated and compared.

To further derive tropism switch-related pattern pairs, three criteria were imposed. First, linear regressions were performed for all candidate pattern pairs – those having significant t-tests and having the same location in the V3 region. The degree of association for each pair was evaluated through slopes from linear regressions between these candidate pairs. To be more precise, each pattern had eight points calculated in eight groups, and the p-values from linear regression were used to evaluate association strength between the eight points of one pattern to another (P < 0.05). Second, the negative slope of the regression line indicated an inverse relationship between the two patterns. Under some circumstances, one mutational pattern could be highly associated with another two patterns. In this case, we added up these two patterns. Third, as a final precaution, to insure that no consensus AAs were identified as pattern pairs in the two-AA case, each AA within the two-AA pattern should be significant between the R5 and X4 dataset. Pairs that passed all three criteria were finally identified as pattern pairs.

## Results

### CM Classifier Evaluation

**Figure 1:**
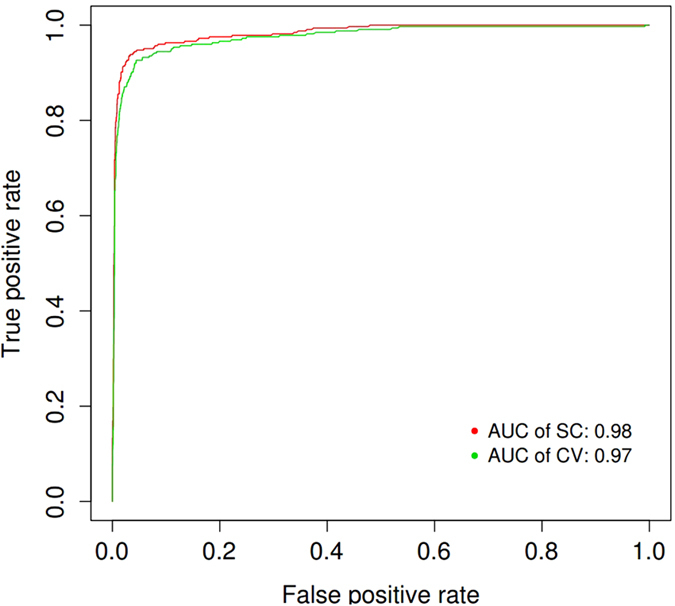
Receiver operating characteristic (ROC) curves of the CM classifier. The ROC values for the self-consistency and cross validation are plotted. The red and green curve represent the ROC curve for self-consistency and ten-fold cross validation for training dataset separately. The legend lists the value of the area under the receiver operating characteristic curve (AUC).

To assess the overall performance of our classifier, we calculated the AUC using the ROCR package^45^. For this classifier, we found AUC values of 0.98 and 0.97 for self-consistency and ten-fold cross validation, respectively (Fig. 1). An accuracy of 95.21% with an MCC of 0.885 (Table 1) for the ten-fold cross validation was achieved.

### Comparison with other Methods in an Independent Dataset

To support our assessment of the performance of the CM classifier, we additionally tested the CM classifier and seven other methods (geno2pheno^17^, hivcopred^16^, dskernel^31^, PSSM^18^, the 11/25 rule^14^, the combined charge rule^46^ and WetCat^15^) using an independent test set comprised of 221 R5 and 91 × 4 V3 sequences extracted from previous studies (see Methods, independent dataset section). As described earlier, these sequences are disjoint from our training dataset.

All comparison methods excepting for the 11/25 and combined charge rule, can be obtained via their respective websites. The 11/25 rule classifies viruses as CXCR4 tropic if a positively charged amino acid is observed in positions 11 or 25 of the V3 sequence. The combined charge rule^46^ integrated net charge rule with charge properties in position 11/25. This rule requires one of the following criteria for predicting CXCR4 coreceptor usage: either 11R/K, 25K, or both; 25R and a net charge of at least 5; a net charge of at least six. For geno2pheno, we chose a default false positive rate (FPR) of 20%, which was suggested by European guidelines for singlicates. Dskernel had three models: dskernel-X4, dskernel-R5, and dskernel-R5 × 4. Hivcopred had two models: Hivcopred (SAAC) and hivcopred (Hybrid). We sorted our results based on the accuracy of each method. As shown in Table 2, the CM classifier did well and exhibited higher accuracy and MCC values than the other methods.

### Pattern Mutation

**Figure 2:**
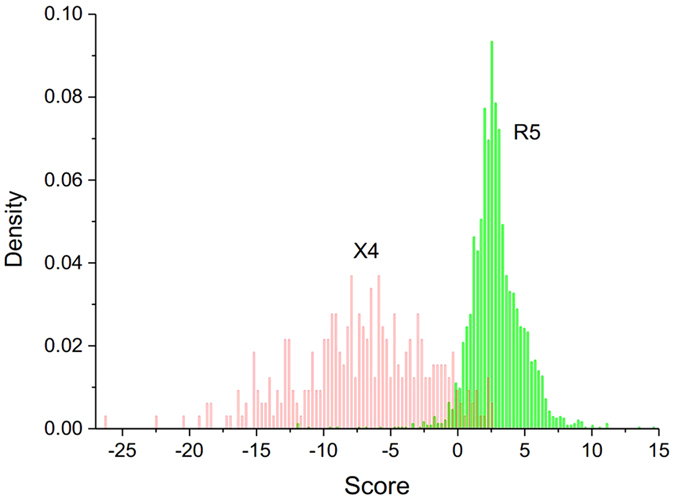
Distribution of the V3 loop sequence scores calculated from the CM classifier. The histogram in red provides a density distribution of the scores for X4-tropic V3 sequences. The density distribution of the R5-tropic sequences scores is shown in green.

Based on Fig. 2, identifying the sign of the sequence score allows us to classify each sequence into these two coreceptor tropisms (Table S2).

**Figure 3:**
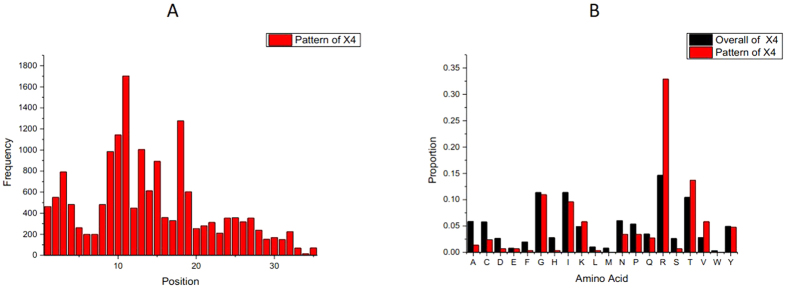
(**A**): Distribution of the positions of X4-tropic conserved patterns. All positions were normalized by aligning them to the consensus sequence. Note that not all unaligned sequences for the V3 loop have a length of 35, and the locating procedure would probably shift the original positions of the unaligned sequence of the V3 loop towards a new position close to the original one. Generally, the higher the peak at the respective position, the more important the position is for coreceptor tropism determination. (**B**): Amino acid compositions of the entire X4 dataset and the X4-tropic conserved patterns. The histogram in black is the proportions of 20 kinds of amino acids for the whole X4 dataset. The histogram in red is the proportions of 20 kinds of amino acids for the X4-tropic specific conserved patterns.

In the first step of pattern analysis, we obtained an overview of the important positions and relevant AAs for tropism switch. Figure 3 shows the position distribution of the X4-tropic conserved patterns and the different AA composition for the whole X4 subset and the conserved patterns of X4. An important note is that the distributions for Pat20-3 and Pat20-4 (Figure S2) were similar to the results for Pat20-2. In this case, we chose to use the succinct Pat20-2 to demonstrate significant positions and AAs for coreceptor switch.

**Figure 4:**
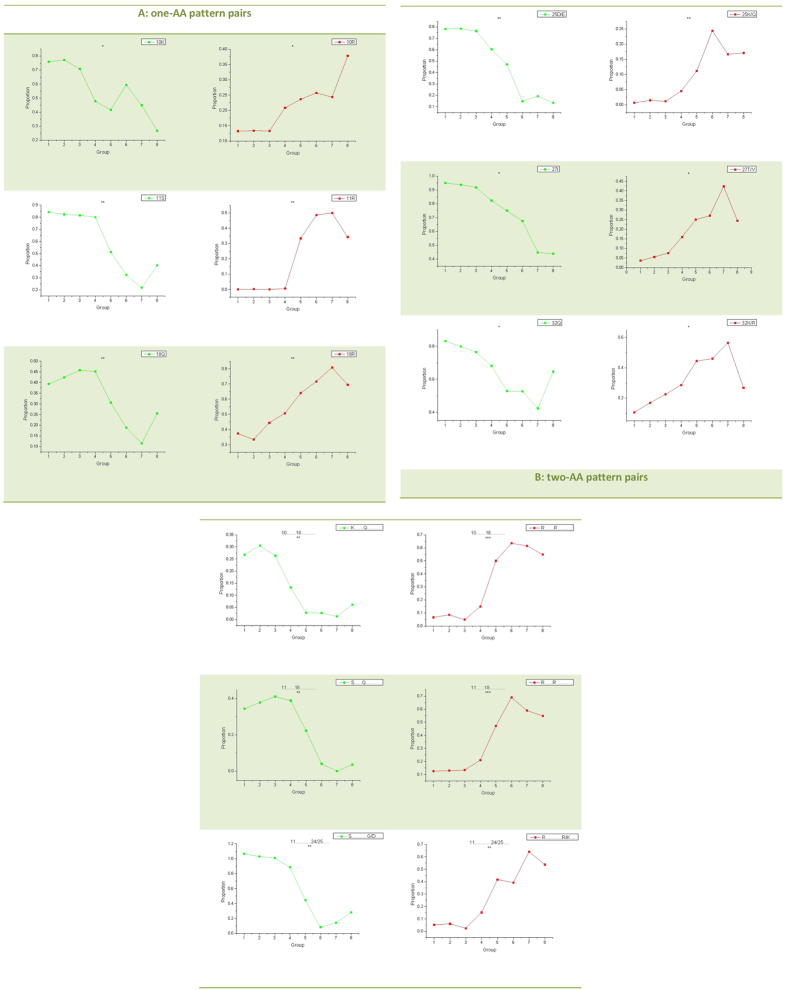
Mutational patterns along with an increasing propensity for the X4 tropism. All lines consist of eight points. The first four points are pattern proportion values in four R5 groups, and the remaining four points are calculated from X4 groups. The proportion of one pattern is the ratio between the count number of the pattern in one group and the group size (count of all sequences in this group). The lines are colored according to the variation tendency of each pattern (green denotes patterns having decreasing proportions from R5 to X4 groups; red denotes patterns that have increasing proportions from the R5 to X4-tropic groups). T-test significance (p < 0.05) was marked by asterisks on top of each line. Significance codes are “0 ‘***’ 0.001 ‘**’ 0.01‘*’ 0.05”. (**A**): one-AA pattern pairs. The pattern symbol including information of AA and position is shown in the top right box. (**B**): two-AA pattern pairs. The pattern symbol is shown in the top right box and the corresponding position information is marked on the top of each line.

As a whole, the positions of X4-tropic patterns are located at discontinuous regions of the V3 loop. Relevant AAs for tropism switch include R in positions 10, 11, 18, and 25; D in position 25; S in position 11; V in position 27; H in position 13; and A in position 22. The highly visible position 11 is the highest peak, in accordance with the 11/25 rule^14^ or the 11/24/25 rule^39^. However, positions 24 and 25 are not prominent. Instead, these positions show the smallest dip, consistent with the results from^35^,^47^ and indicating higher relevance of position 11 compared to 24/25 in coreceptor switch. The subsequent highest peak is located in position 18, which corresponds to the core of the crown of the V3 loop. This area is called an arch and is a conserved structural element^48^ used for binding to the coreceptor’s second extracellular loop (ECL2) region^49^. There are two main amino acid combinations in this tip motif: Gly-Pro-Gly-Arg/Gln (GPGR/Q). Evidence suggests that amino acid substitutions from the conserved V3 loop crown motif GPGQ to GPGR are associated with the X4 genotype^37^,^49^,^50^. From pattern searching the crown motif, a ‘peak’ was found in position 15, which is just a consensus residual G across the whole dataset. Another peak is located at positions 10 and 9 of the V3 loop. The presence of R (Fig. 3B) is in accordance with these peaks. Apart from these peaks, position 13 is also an outstanding position for X4-tropic conserved patterns. The corresponding AA change for position 13 is a decline in H (see Table S4, Sheet1), which is consistent with the decreased proportion of H in Fig. 3B. As to amino acid A, a significant decline after tropism switch could be seen from Fig. 3B and Table S4, Sheet1. Pattern mutations across the eight groups regarding other significant AAs in Fig.3B and positions were shown in Fig. 4.

Detailed pattern pair identification was performed across eight groups following identification of important AAs and positions. In total, nine pairs of mutational patterns were identified and associated with increasing propensity for X4 tropism. Two mutational patterns for each pair were separately shown on the left (green color) and right (red color) part in Fig. 4. These nine mutational pattern pairs can be divided into two main classes: six pairs of one-AA pattern mutations and three pairs of combinations of two-AA mutations (Fig. 4A,B, respectively). No combinations of mutations in three or four amino acids were found through the Pat20-3 and Pat20-4 search (data not shown), indicating that the top number of combinations of amino acid alternations is two.

Four one-AA pattern pairs including 11S to 11R, 18Q to 18R, 25D/E to 25K/Q, and 32Q to 32K/R are charge rule related pattern mutations (Fig. 4A). Pattern changes in positions 11 and 25 are in accord with the 11/25 charge rule. All these four pattern pairs increase net charge of the V3 loop, but have different degrees of impact. Compared with average proportion of 11R in R5, the average proportion of 11R is 41% higher in X4. After 11R, charge degree increases due to one-AA mutations ranked in the order of 18R (30%), 25K (15%), 32R (13%) and 32K (11%). An important note is that the increasing amount of one pattern does not exactly match the decreasing amount of its partner. Of the above four pairs, only the increase in 11R matches the decrease in 11S, equalizing the mutation degree. Another positively charged AA pair in position 10 is 10R to 10K (Fig. 4A), which is consistent with the position peak in Fig. 3a. These two-AA are very similar in their charge properties but differ significantly in their polar and acidic properties. The last pair in Fig. 4A is 27I to 27T/V. In structure-based methods for determining virus tropisms, hydrophobicity is usually relevant (25, 51). Around 33% lower average proportion of 27I in X4 groups than in R5 groups led to a loss of hydrophobicity, despite the increased 10% proportion of its hydrophobic partner, V.

Based on the pattern pairs in Fig. 4A, there would mathematically be 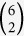 kinds of cases for two-AA pattern pairs. However, not all combinations of candidate positions could co-mutate with each other and only three pairs of combined AA mutations were found and shown in Fig. 4B: 10K18Q to 10R18R; 11S18Q to 11R18R; and 11S [24|25][G|D] to 11R[24|25][R|K]. Different degrees of change were also found in these pairs by calculating the average pattern proportions in R5 and X4 groups. For example, there was a high degree increase (49%) in 10R18R but only a 21% drop in 10K18Q. Similarly, 11R18R had a higher increase (43%) than the decrease (31%) in 11S18Q.

## Discussion

This study developed a new but effective genotypic model for predicting HIV-1 coreceptor usage. To do this, we transformed R5-tropic and X4-tropic datasets into two different numerical CMs. We assumed the corresponding CMs represented the features of the datasets from which they came. Following this assumption, the distances of a new V3 sequence from the R5 “family” dataset and the X4 “family” dataset could be measured by these two CMs. In theory, any sequence should have a short distance and high probabilistic value if it had biological functions similar to those of a family, i.e., an R5-tropic sequence would obtain a higher score on R5-M than X4-M. Similar performances in self-consistency (0.98 AUC) and ten-fold cross validation (0.97 AUC) suggest the reliability and robustness of our classifier. Additional support for our model is inferred from the comparison with six other classifiers using an independent dataset. Precisely, our CMs found a 56% higher MCC value than the classical coreceptor prediction approach – the 11/25 rule – which was too simple to capture all significant features. Since we incorporated charge rules (11/25 and net charge) and a dividing factor to weight each AA in developing CMs, a 15% higher MCC compared to PSSM and a 13% higher MCC compared to geno2pheno were found. Moreover, all dual-tropic sequences in independent dataset were correctly predicted (data not shown). The strong generalization ability of our classifier confirmed the correct design of our algorithm. One limitation of the classifier should be taken into account: our classifier did not show the same performance for different subtypes (Table S5). Accuracies for subtypes A, B, C and 01_AE were above 90%, whereas subtype D had an accuracy of 84%. The relative low accuracy of subtype D may be due to a few reasons. First, there was a relatively low identity of subtype D, which suggested that sequences from this group have low similarity with each other. Second, we used a relatively small training set compared to the training set of subtype B. In order to address issues possibly arising from non-B subtypes, we developed two subtype specific classifiers (detailed on our web server) based on the algorithm described in the methods for subtype C and subtype D. These subtypes are important due to their rapid spread and large representation in worldwide infections. An accuracy of 97.02 and accuracy of 97.48 were found in the cross validation of subtype C and subtype D specific classifiers (Table S6). With the increase in sequence number, our classifiers would achieve better performance. Another limitation is the size of the independent dataset, which is much smaller compared to the training dataset. To compensate for this and estimate how accurately our classifier would perform in practice, we performed a 10-fold cross-validation. This served as an impartial way to evaluate the performance in real problems despite the small size of the independent dataset.

The CM classifier score not only offers a way to measure the propensity for X4-coreceptor usage for each V3 loop sequence – as illustrated by score decrease from R5- to X4-tropic sequences (see Fig. 2) – but also allows the possibility of deriving pattern mutations and investigating the mechanism along the R5 to X4 tropic shift. Based on the scores, our method derived 18 mutational patterns associated with increasing propensity for CXCR4 tropism. A partner for each pattern was also identified by association test.

Several points on mutation pattern effects on tropism switch should be raised. The first point deals with the effects of charge distribution on the V3 loop flexibility, which is associated with coreceptor usage^51^.We know that arginine in position 3 is a consensus AA across the V3 region of HIV. Charge rule related mutational patterns located in positions 11, 18, 25 and 32 of the V3 loop (Fig. 4) had higher proportions in the X4-tropic groups. Starting from position 11, four pattern pairs were identified at every seventh consecutive position. Position 18 acted as an inflection point, with 3R and 11R in the first half of the loop and 25K and 32R/K in the other half. In this case, there were four positively charged AAs symmetrically distributed across the two half-loops (position 3 close to 25 and position 11 close to 32). To probe the structure variations caused by pattern mutation, we used modeller^52^ to separately simulate structures of a R5- and X4-tropic sequence. The X4-tropic sequence contained all mutational patterns identified through our pattern screen, whereas the R5-tropic sequence contained none of them. Here, the crystal structure^53^ of V3 loop in HIV-1 gp120 core (PDB id: 2b4c) was used as a modelling template and twenty structures were built for each sequence. A single, best structure was selected based on DOPE scores from the twenty simulated structures. For the two best structures, we calculated the distance between Ca on the two sides (residuals 1–17 and 19–35) of the V3 loop. This side-to-side distance was compared between the R5 and X4 loop with a paired t-test which revealed a wider loop of X4 relative to R5 (p = 0.03). A visual representation of the two simulated loop structures and corresponding sequences (Figure S3) also suggested the structural difference. In other words, not only does net charge influence coreceptor tropism, but distribution of charge residuals likely influences coreceptor tropism as well. Similar to charge effect on loop structure, hydrophobic pattern mutations like 27I to 27T/V (Fig. 4) and decreased I in positions 2, 12, 27 and 30 (Table S4, Sheet3) could also lead to a more open V3 loop due to increasing hydrophilicity and polarity. More convincing studies on V3 loop structures should be performed to evaluate tropism associated structural changes caused by the sequence pattern mutation.

Another point is different amounts of change in patterns suggested different degrees of impact on coreceptor switch. Generally, a greater level of change suggested a higher weight in coreceptor switch. No pattern showed a complete (0 to 1; 1 to 0) switch along with the tropism shift. In addition to levels of change, absolute proportion values of one pattern in one group provided us more specific information to explore the coreceptor switch mechanism. For example, both 11R and 18R showed high, increased change, 41% and 30% respectively. However, the initial average proportion of 11R in R5 groups was 0%, whereas the average proportion of 18R in R5 groups was 41%. Despite both showing similar increased levels, 11R and 18R made different contributions to coreceptor switch: R in position 11 suggested a switched tropism, whereas R showed in position 18 did not. Both the level of change and the absolute proportion value of each pattern should be taken into account when studying pattern role orientation in coreceptor switch.

One special one-AA pattern pair is 10K to 10R which are both positively charged but differ in their molecular weight, polar and acidic properties. In a previous study, higher infectivity of HIV has been found because of a K to R alternation in positions 9, 10 and 11^54^. Based on our results, no significant alternations of K to R were found in positions 9 and 11 (Fig. 4 and Table S4, Sheet1); however, we did find a significant K to R alternation in position 10. Despite the similarity in charge between K and R, this AA switch was significantly associated with tropism switch and also should be considered when predicting HIV phenotype.

As we know, the loss of N-linked glycosylation sites is associated with the coreceptor switch^55^. The location of potential N-linked glycosylation sites are determined by specific motifs instead of fixed position of V3 loop. As a result, the loss of N-linked glycosylation sites could not be caught by our one-AA mutational pattern search precisely. Instead, we used N-Glycosite tool developed by Zhang, M. *et al.*^56^ to identify N-linked glycosylation sites in V3 amino acid sequences from eight groups. A significant difference (p = 0.04, t-test) of N-linked glycosylation sites between R5-tropic and X4-tropic sequences was consistent with previous studies and suggested the correctness of our scoring system.

Aside from one-AA mutational patterns, three pairs of two-AA mutational patterns were also recognized out of a possible 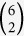 pairs (Fig. 4). The low number of two-AA pairs indicated that the choice of co-mutational AAs by HIV were not accidental. An important note is that amounts of change in two-AA patterns did not necessarily match the change amounts of the corresponding one-AA patterns (Table S4, Sheet3 and Sheet4). Take 11S18Q as an example. As shown in Table S4, the average decreasing proportion of 11S18Q is 31% from R5 to X4. This does not suggest a 31% drop both in 11S and 18Q individually. Any two-AA pattern in Fig. 4 could suggest a co-mutation of two one-AA patterns. The decreasing change of two-AA pattern 10K18Q is 21%, which was the same with the decreased degree in 18Q. This suggests that every single disappearance of Q in position 18 is bound with the disappearance in 10K. On the other hand, a 25% lower proportion was found in average for 10K in X4 groups. In this case, disappearance in 10K does not necessarily represent a binding disappearance in 18Q. In fact, only 21 of 25% (84% in total) of 10K were involved in co-mutation of 10K18Q. Similarly, an increase by 49% of 10R18R – the partner of 10K18Q – only partially replaces 10K18Q. 10R18R accounts for the decreased 10K18Q and also replaces other amino acids leading to a high average proportion, 57%, in X4 groups.

Our findings greatly aided in monitoring coreceptor switch and understanding the mechanism of coreceptor switch through identifying pattern mutations. Different significant pattern pairs with different levels of change suggested the flexibility of the V3 region and the complexities of tropism switch mechanisms. Given the extensible application of next generation sequencing technology, an abundance of sequences can be readily analyzed to solve different problems. Our method not only provides a classification scheme for HIV viral strains with differing tropism, but could generalize to a broader method for solving other pattern mutation-related problems.

## Additional Information

**How to cite this article**: Shen, H.-S. *et al.* HIV coreceptor tropism determination and mutational pattern identification. *Sci. Rep.* **6**, 21280; doi: 10.1038/srep21280 (2016).

## Supplementary Material

Supplementary Figure S1, S2, S3; Table S1, S3,S5, S6

Supplementary Dataset 1

Supplementary Dataset 2

Supplementary Dataset 3

Supplementary Dataset 4

Supplementary Dataset 5

Supplementary Dataset 6

## Figures and Tables

**Table 1:** Validation of the CM classifier.

Method	Sensitivity	Specificity	Accuracy	MCC
CM (Self-Consistency)	94.15	96.09	95.84	0.903
CM (Cross Validation)	92.92	95.54	95.21	0.885

**Table 2:** Performance of the CM classifier and other methods for an independent dataset.

Method	Sensitivity	Specificity	Accuracy	MCC
CM	91.21	95.02	94.54	0.863
dskernel-X4	83.51	96.38	92.63	0.806
Geno2pheno(FPR = 20)	97.83	76.92	91.73	0.764
Hivcopred (SAAC)	88.23	91.20	90.33	0.795
Hivcopred (Hybrid)	88.23	91.20	90.33	0.795
PSSM	81.31	93.21	89.74	0.751
Combined charge rule	74.73	94.21	88.46	0.702
11/25	52.74	96.83	83.97	0.552
dskernel-R5	26.37	100.00	78.52	0.390
dskernel-R5 × 4	13.18	99.09	74.03	0.240
WetCat	83.51	69.23	73.40	0.533

The results are sorted according to their accuracy. The CM classifier result is shown in bold.
