# Supplementary material for: HIV coreceptor tropism determination and mutational pattern identification
Source: Sci Rep. 2016 Feb 17;6:21280. doi: 10.1038/srep21280 (PMC4756667; doi:10.1038/srep21280)
Supplement: Supplementary Figure S1, S2, S3; Table S1, S3,S5, S6 [file srep21280-s1.pdf]

HIV coreceptor tropism determination and mutational pattern identification

Hui-Shuang Shen[1], Jason Yin[2], Fei Leng[3], Rui-Fang Teng[4], Chao Xu[5], Xia-Yu Xia[6], Xian-Ming Pan[*]

1. The Key Laboratory of Bioinformatics, Ministry of Education, School of Life Sciences, Tsinghua University, China. Email: shenhs11@mails.tsinghua.edu.cn
2. Department of Biostatistics, Saw Swee Hock School of Public Health, National University of Singapore, Singapore. Email: jason_yin@nuhs.edu.sg
3. The Key Laboratory of Bioinformatics, Ministry of Education, School of Life Sciences, Tsinghua University, China. Email: 512793853@qq.com
4. The Key Laboratory of Bioinformatics, Ministry of Education, School of Life Sciences, Tsinghua University, China. Email: trf14@mails.tsinghua.edu.cn
5. The Key Laboratory of Bioinformatics, Ministry of Education, School of Life Sciences, Tsinghua University, China. Email: cxu12@mails.tsinghua.edu.cn
6. The Key Laboratory of Bioinformatics, Ministry of Education, School of Life Sciences, Tsinghua University, China. Email: xiaxiayu.thu@hotmail.com

* Correspondence to: Xian-Ming Pan, The Key Laboratory of Bioinformatics, Ministry of Education, School of Life Sciences, Tsinghua University, Beijing, 100084, China.

Tel: +86-10-62792827, Fax: +86-10-62792827.

Email: pan-xm@mail.tsinghua.edu.cn

Figure S1. Flow chart for data filtering

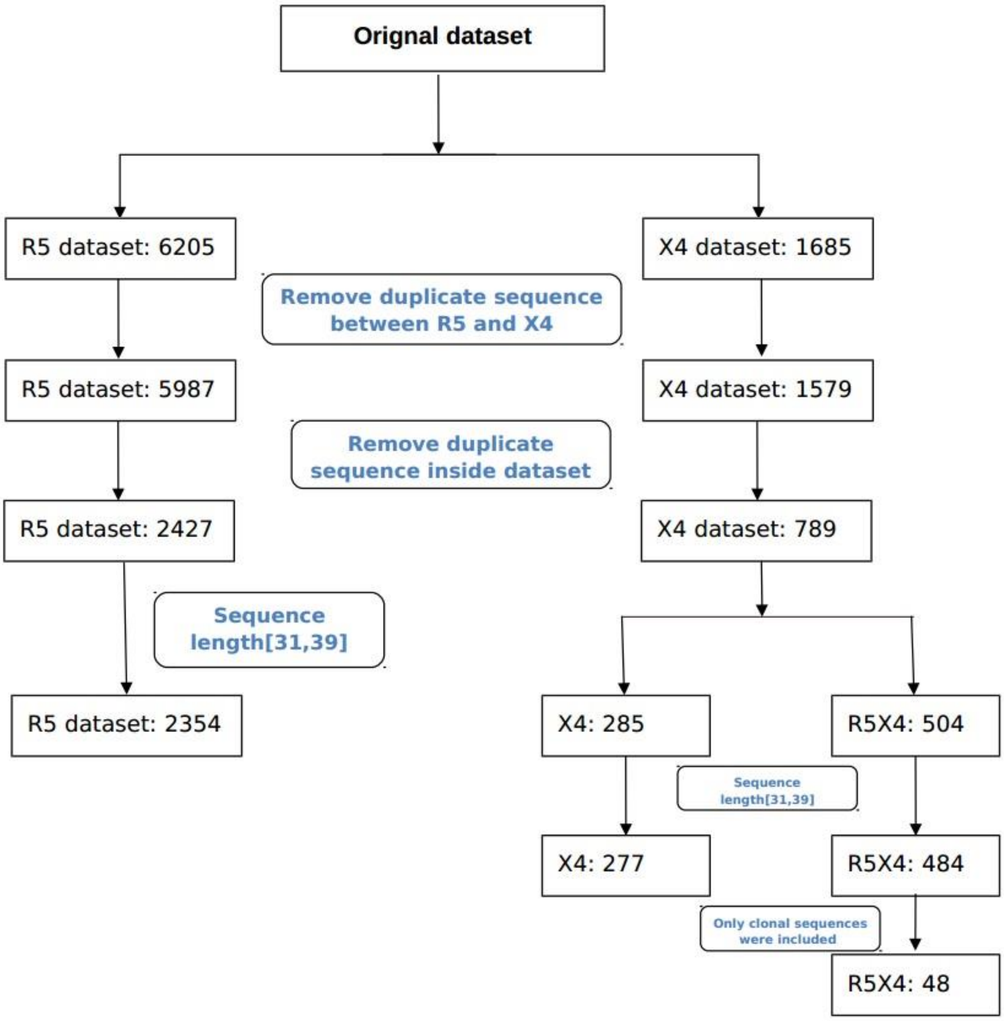

Figure S2. Distribution of positions for X4-tropic conserved patterns in Pat20 (3) and Pat (4).

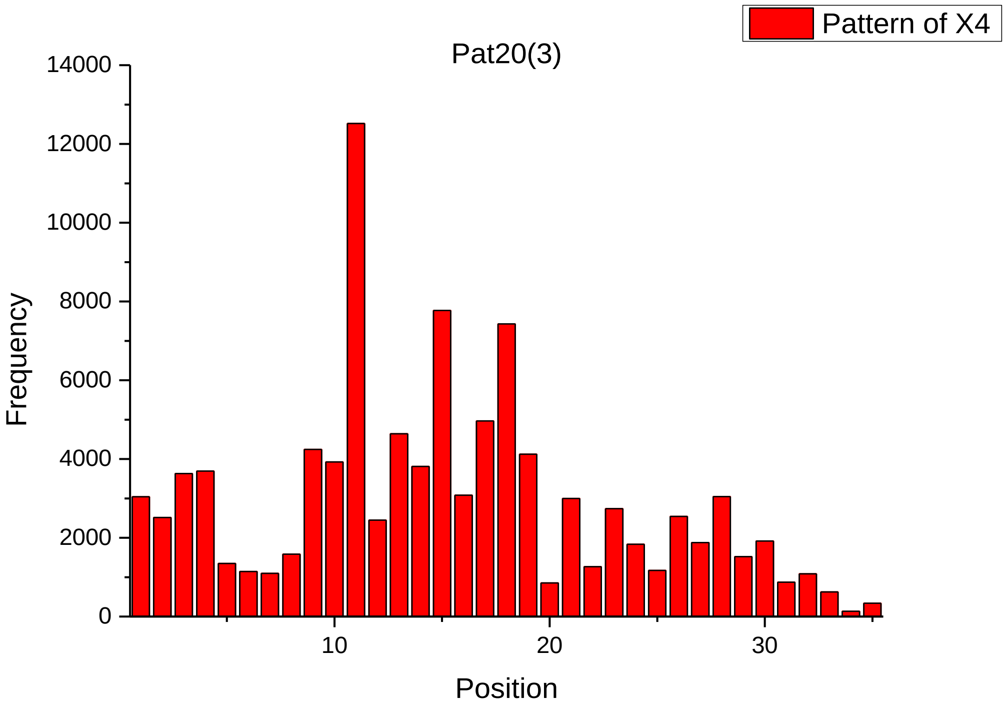

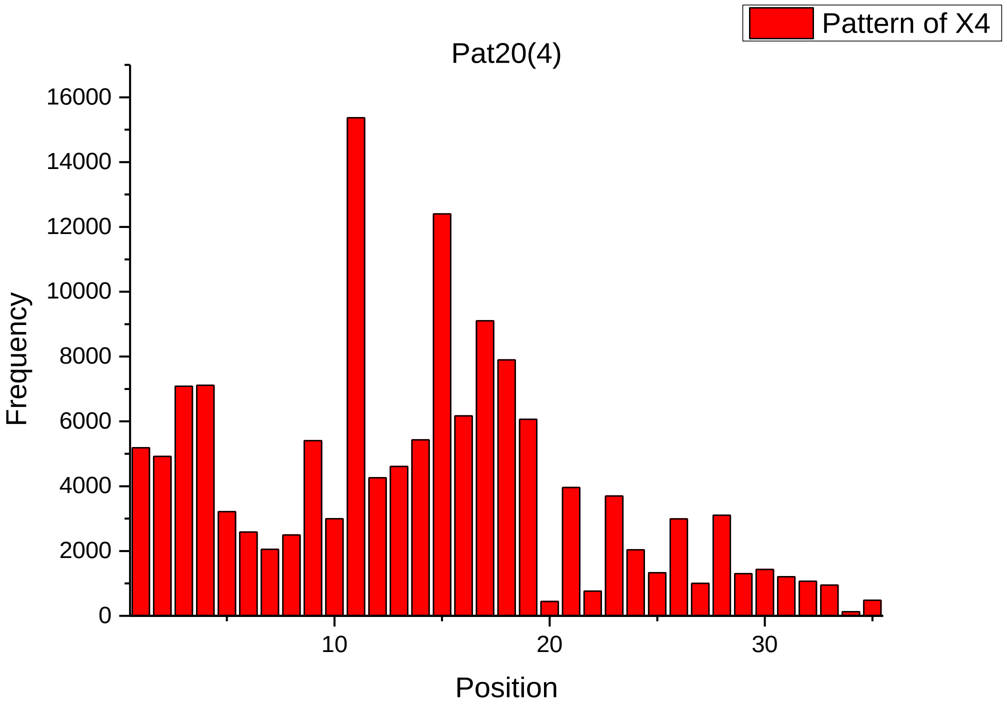

Figure S3. Simulated structures for R5 (CTRPNNNTRKSIHIGPGQAFYATGDIIGDIRQAHC) and X4 (CTRPNNNTRRRITIGPGRAFYATGKITGDIRRAHC) sequences. The left image in part 'a' showed the electrostatic potential distribution of R5 and the right one was for X4. The red, white and blue represented negative potential, near neural, and positive potential, respectively. Part 'b' showed the aligned structures of R5 (hot pink) and X4 (light blue). The average side-to-side distance of X4 is significant larger than that of R5 (p=0.03).

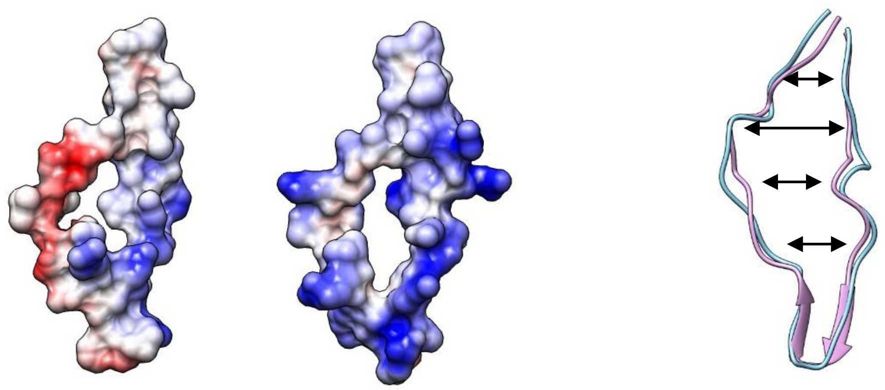

Table S1. Frequency and dividing factor for each amino acid in R5 and X4 dataset.

R5			X4		
AA	frequency	dividing factor	AA	frequency	dividing factor
C	4682	0.0568	N	674	0.0593
W	141	0.0017	C	647	0.0569
N	6996	0.0849	L	117	0.0103
B	703	0.0085	B	189	0.0166
P	4786	0.0581	V	316	0.0278
E	892	0.0108	Q	388	0.0341
V	1078	0.0131	P	602	0.0529
M	498	0.0060	I	1273	0.1119
K	2353	0.0286	F	221	0.0194
R	9313	0.1130	R	1637	0.1439
G	9750	0.1183	M	90	0.0079
H	2755	0.0334	K	548	0.0482
D	2872	0.0349	H	312	0.0274
L	484	0.0059	G	1271	0.1117
I	10596	0.1286	W	36	0.0032
Q	3107	0.0377	T	1168	0.1027
A	5805	0.0705	A	655	0.0576
T	8006	0.0972	S	293	0.0258
Y	2769	0.0336	Y	553	0.0486
S	2633	0.0320	E	88	0.0077
F	2171	0.0264	D	297	0.0261

Table S3. Eight groups with gradually decreasing scores along the R5 to X4 transition. The first four groups belong to the R5 dataset, and the other four groups belong to the X4 dataset.

Coreceptor	Score Interval	Group Size
R5	(5,15]	303
R5	(3,5]	681
R5	(2,3]	702
R5	(0,2]	576
X4	(-4,0]	72
X4	(-7,-4]	74
X4	(-10,-7]	78
X4	(-27,-10]	82

Table S5. CM performance for different subtypes.

Subtype	R5.Seq No.(identity)	X4.Seq No.(identity)	Identity	Sensitivity	Specificity	Accuracy	MCC
A	145(77.96)	6(59.04)	76.83	100.00	99.31	99.40	0.993
B	1229(78.67)	130(64.67)	76.53	93.85	96.66	96.30	0.905
C	472(81.31)	50(64.07)	79.12	88.00	98.94	97.55	0.875
D	137(70.75)	60(60.87)	63.77	100.00	81.75	84.07	0.831
01_AE	134(81.82)	54(65.95)	75.40	100.00	88.81	90.23	0.894

Table S6. Validation for subtype C and subtype D specific classifiers.

	**Sensitivity**	**Specificity**	**Accuracy**	**MCC**
Self-consistency of **Subtype C**	93.75	98.09	97.54	0.919
10-fold cross validation of **Subtype C**	92.71	97.65	97.02	0.905
Self-consistency of **Subtype D**	94.37	99.31	98.68	0.938
10-fold cross validation of **Subtype D**	94.37	97.93	97.48	0.924
